# Mass Spectrometric Detection of Bacterial Protein Toxins and Their Enzymatic Activity

**DOI:** 10.3390/toxins7093497

**Published:** 2015-08-31

**Authors:** Suzanne R. Kalb, Anne E. Boyer, John R. Barr

**Affiliations:** Centers for Disease Control and Prevention, 4770 Buford Hwy NE, Atlanta, GA 30341, USA; E-Mails: skalb@cdc.gov (S.R.K.); aboyer@cdc.gov (A.E.B.)

**Keywords:** anthrax, anthrax lethal factor, botulinum neurotoxin, botulism, mass spectrometry

## Abstract

Mass spectrometry has recently become a powerful technique for bacterial identification. Mass spectrometry approaches generally rely upon introduction of the bacteria into a matrix-assisted laser-desorption time-of-flight (MALDI-TOF) mass spectrometer with mass spectrometric recognition of proteins specific to that organism that form a reliable fingerprint. With some bacteria, such as *Bacillus anthracis* and *Clostridium botulinum*, the health threat posed by these organisms is not the organism itself, but rather the protein toxins produced by the organisms. One such example is botulinum neurotoxin (BoNT), a potent neurotoxin produced by *C. botulinum*. There are seven known serotypes of BoNT, A–G, and many of the serotypes can be further differentiated into toxin variants, which are up to 99.9% identical in some cases. Mass spectrometric proteomic techniques have been established to differentiate the serotype or toxin variant of BoNT produced by varied strains of *C. botulinum*. Detection of potent biological toxins requires high analytical sensitivity and mass spectrometry based methods have been developed to determine the enzymatic activity of BoNT and the anthrax lethal toxins produced by *B. anthracis*. This enzymatic activity, unique for each toxin, is assessed with detection of the toxin-induced cleavage of strategically designed peptide substrates by MALDI-TOF mass spectrometry offering unparalleled specificity. Furthermore, activity assays allow for the assessment of the biological activity of a toxin and its potential health risk. Such methods have become important diagnostics for botulism and anthrax. Here, we review mass spectrometry based methods for the enzymatic activity of BoNT and the anthrax lethal factor toxin.

## 1. Introduction

Accurate microorganism identification is critical for correct diagnosis of disease, treatment of bacterial infection, and prevention of further bacterial disease outbreaks. Additionally, bacterial identification is important for microbial forensics, criminal investigations, and environmental studies. Recently, bacterial identification by mass spectrometry has emerged as a popular technique for accurate microorganism identification. First proposed in 1975 [1], the technique has recently employed the use of matrix-assisted laser-desorption time-of-flight (MALDI-TOF) mass spectrometry for detection. This technique currently uses a 12–24 h culture in which a small amount of the bacterial colony is transferred onto a stainless steel MALDI plate followed by the addition of a matrix compound which lyses the cell walls thereby extracting protein, and aids in ionization of the sample. The mixture is then analyzed by TOF mass spectrometry. A bacterial protein “fingerprint” is obtained consisting of peaks corresponding to small soluble proteins from the bacteria. Each fingerprint pattern is unique, and with the addition of statistical methods and database searching, an accurate bacterial identification can be made.

This technique has been successful for accurate and rapid identification of bacteria from cultures, allowing for disease diagnosis, treatment, and prevention as mass spectrometric analysis is quite sensitive and specific. However, there exist bacteria such as *Bacillus anthracis* and *Clostridium botulinum*, with which the health threat posed by these organisms is not the organism itself, but rather the protein toxins produced by the organisms. For instance, not all *Clostridium botulinum* produce botulinum neurotoxins, and botulinum neurotoxins are produced by other *Clostridia*, so toxicity is dependent upon the existence of the toxin itself and not the organism. *B. anthracis* produces two binary toxins including lethal toxin, and edema toxin whereas *C. botulinum* produces botulinum neurotoxin (BoNT). Although these protein toxins are both produced by bacteria, the functions of the toxins are different and unique.

**Figure 1:**
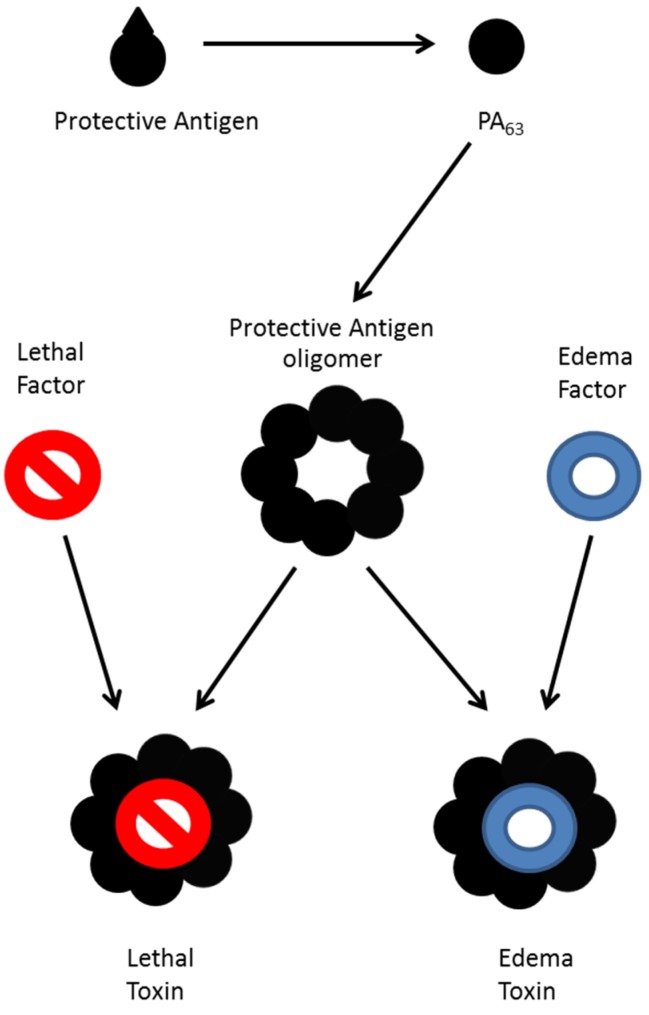
Formation of toxin from *B. anthracis*. Protective antigen is cleaved and oligomerizes, then binds lethal factor to form lethal toxin, edema factor to form edema toxin, or both to form a mixed toxin.

*B. anthracis* produces lethal factor (LF), edema factor (EF), and protective antigen (PA) [2]. Protective antigen is an 83 kDa protein which is cleaved into a 63 kDa portion (PA63) which forms heptamers and octamers that binds to cell surface receptors [3,4,5]. The PA63 oligomer also combines with three to four molecules of lethal factor as seen in Figure 1 to form lethal toxin (LTx) or edema factor to form edema toxin (ETx) or may bind both to form a mixed toxin. LF is a zinc-dependent endoprotease which cleaves mitogen activated protein kinase kinase (MAPKK) [6], and EF a calcium and calmodulin-dependent adenylyl cyclase that converts ATP to cyclic AMP [7]. Both toxins work together to cause disruption of the immune system, septicemia, hemorrhage, and shock, which can lead to death [8,9,10].

**Figure 2:**
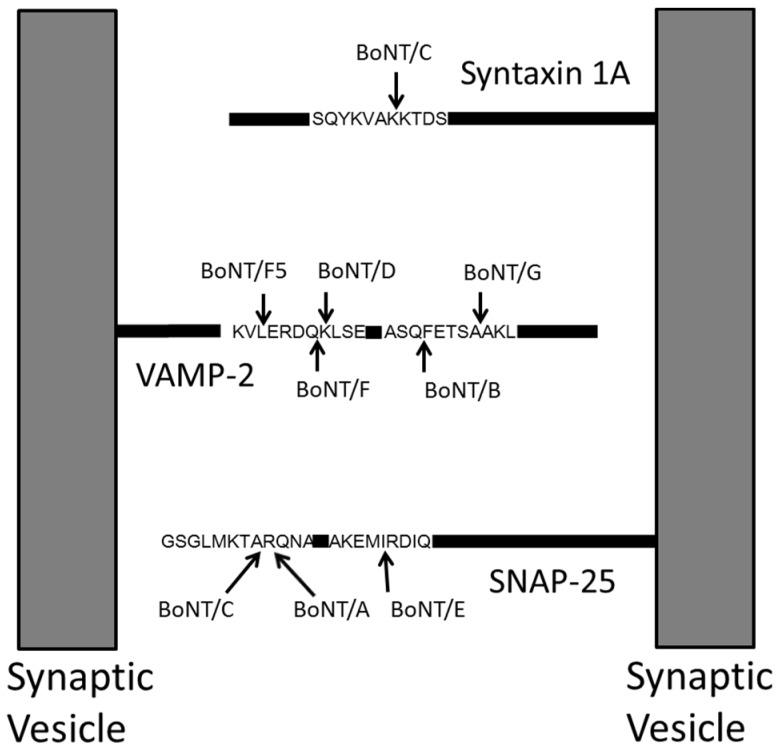
BoNT cleaves surface proteins of synaptic vesicles. BoNT/A, /C, and /E cleave SNAP-25, BoNT/B, /D, /F, /F5, and /G cleave VAMP-2, and BoNT/C cleaves syntaxin 1A.

*C. botulinum* produces botulinum neurotoxin (BoNT), which is currently categorized into seven serotypes labeled A–G, based on their response to antisera. BoNT is a 150 kDa protein composed of a heavy chain of approximately 100 kDa and a light chain of about 50 kDa. The heavy chain binds to receptors on the surface of neurons and the light chain cleaves proteins necessary for nerve signal transmission. BoNT/A, /C, and /E cleave SNAP-25 (synaptosomal-associated protein) [11,12,13,14,15,16], and BoNT/B, /D, /F, and /G cleave synaptobrevin-2 (also known as VAMP-2) [17,18,19,20,21,22] as seen in Figure 2. Identification of the serotype of BoNT is important because each serotype is neutralized by a different antiserum. BoNTs can also be categorized below the serotype level, known as subtype differentiation. Different strains of *C. botulinum* can produce different subtypes, or toxin variants (neurotoxin protein), and some of the neurotoxins manufactured by different strains have as few as a single amino acid difference, or 0.08% difference. Differentiation of the BoNT subtype is important to forensic and epidemiologic investigations endeavoring to ascertain the toxin’s source, its spread in a botulism incident, and commonality/differences in concurrent botulism outbreaks. Additionally, the varied subtypes potentially have differences in their virulence and their ability to be neutralized by antiserum.

Because *B. anthracis* and *C. botulinum* produce proteins with enzymatic activities that are detrimental to the health of animals or people exposed to the toxins, it is important to determine not solely the toxin’s presence, but also to assess its enzymatic activity. A study of the enzymatic function of the toxins provides an accurate measurement of the health threat of these toxins. In more recent years, this assessment has been successfully reported using a number of *in vitro* methods. In this work, we review mass spectrometry based methods which determine the enzymatic activity of BoNT and the anthrax lethal factor toxin produced by *B. anthracis*. This enzymatic activity is unique for each toxin and in the case of BoNT, exclusive for each toxin serotype. The enzymatic activity of all BoNT and anthrax toxins, are determined with detection of the toxin-induced cleavage of strategically designed peptide substrates by MALDI-TOF mass spectrometry. Both the BoNT and the anthrax lethal factor assays can be run as a qualitative or quantitative assay depending on the need of a particular public health investigation or research study. In this manuscript, the examples provided show the qualitative assay in the case of BoNTs and quantitative assay for anthrax protein toxins. These enzymatic activity measurements in combination with mass spectrometric amino acid sequencing in the case of BoNT yield information needed to diagnose, treat, and prevent botulism and anthrax toxemia.

## 2. Mass Spectrometric Detection of Amino Acid Sequence of Botulinum Neurotoxin

**Figure 3:**
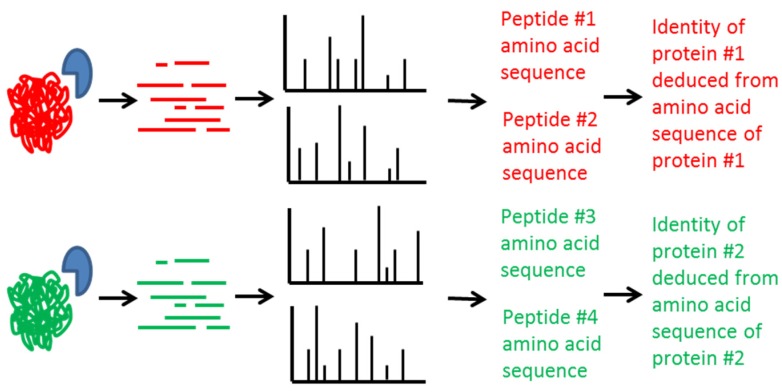
Graphical representation of the use of proteomics methods to determine the identity of BoNT via deduction of its amino acid sequence.

Mass spectrometry can be used to determine the presence or absence of any protein. The seven known serotypes of BoNT are 64% or less identical at the amino acid sequence level. The toxins from different serotypes contain hundreds of amino acid differences and can be differentiated from each other through determination of their amino acid sequences as seen in Figure 3. The toxins are first digested with an enzyme such as trypsin which breaks the protein into many smaller peptides. The peptides are then fragmented inside the mass spectrometer to produce MS/MS which are specific for the amino acid sequence of the peptide. By knowing the amino acid sequence of a peptide originating from the toxin, the amino acid sequence and hence the identity of the toxin can be determined.

Mass spectral analysis of BoNT/A1 and /B1 of purified neurotoxin was first reported by van Baar and colleagues in 2002 [23]. The toxin proteins were digested with trypsin and the resultant peptides were analyzed by MALDI-TOF-MS in order to obtain a peptide mass fingerprinting (PMF) map. Sequence coverage of greater than 35% for BoNT/A1 and 25% for BoNT/B1 was reported using this technique. In 2004, this work was extended to incorporate four additional serotypes: BoNT/C, /D, /E, and /F [24]. Mass spectrometric evidence was reported for 11% sequence coverage of BoNT/C, 49% of BoNT/D, 30% of BoNT/E3, and 53% of BoNT/F1. As these proteins are 36% or more dissimilar, the sequence coverages were sufficient to identify the protein as BoNT and to differentiate the serotype of BoNT for all six serotypes.

BoNT from botulism outbreaks can be present in foods and clinical samples, including serum, gastric, and stool, which contain relatively low toxin levels in the presence of high levels of matrix proteins. Therefore, for botulism outbreak investigations, identification and differentiation of BoNT in a complex matrix is important. Mass spectrometric detection of BoNT/A, /B, /E, and /F (the four serotypes associated with human cases of botulism) in bacterial culture supernatants was reported by Klaubert *et al*. in 2009 [25]. The bacterial culture supernatant was digested with pepsin at pH 2. The BoNT was protected from digestion in this acidic condition by its neurotoxin associated proteins (NAPs) while the matrix proteins were digested to small peptides. Following removal of the unwanted peptides with molecular weight cut-off filters, the retained proteins (consisting of mostly BoNT and its NAPs) were digested with trypsin, separated by 2D nano-LC using ion exchange chromatography and reversed phase chromatography, and analyzed by nano LC-ESI MS/MS. Following database searching, BoNT from bacterial culture supernatants of the four serotypes could be detected and differentiated despite the low sequence coverage (less than 10%) [25].

Alternatively, our laboratory demonstrated that immunoaffinity purification can be used to detect BoNT in the presence of a complex matrix. The use of antibodies to BoNT allows for enrichment of the toxin from food [26,27,28] or bacterial culture supernatant [27,29,30]. Using this process, both BoNT/A [26,27,28,29] and BoNT/B [27,30] were identified and differentiated in complex matrices. This process yielded sequence coverage between 65% and 98%, ensuring correct identification and differentiation of these toxins. These reported techniques use antibodies which are serotype-specific for toxin extraction; however, we also reported development and discovery of an antibody that was able to bind all BoNT/A, /B, /E, and /F [31]. In theory, this antibody could be used for isolation of any BoNT associated with human cases that are present in a complex matrix.

Differentiation of BoNT beyond the serotype level at the subtype/toxin variant level can be important for forensic or epidemiological purposes. Amino acid identity increases to between 70%–97.5% at the subtype level and greater than 97.5% at the toxin variant level. Therefore, high sequence coverage is critical to differentiate BoNT beyond the serotype level. Mass spectrometric differentiation of BoNT below the serotype level was first reported by our laboratory in 2005 in which BoNT/A1 and /A2 subtypes were extracted from spiked milk using serotype-specific antibodies to BoNT/A [26]. Following tryptic digestion, LC-MS/MS analysis, and database searching, the toxins were differentiated as either BoNT/A1 or /A2 with sequence coverages of 65%–70% [26]. Further subtype differentiation of BoNT/A has recently been accomplished with low toxin levels in additional complex matrices such as crude culture supernatants, food, and environmental samples [29].

Subtype differentiation of BoNT/B was first reported by our laboratory in 2010; BoNT/B1 and /B4 were extracted from culture supernatants with immunoaffinity purification, digested with trypsin and sequenced with MS/MS analysis by MALDI-TOF [31]. Additionally, differentiation of five BoNT/B subtypes, BoNT/B1 through /B5, extracted from culture supernatants was reported in 2012 using mass spectrometric techniques [30]. In this work, high sequence coverages (66%–77%) permitted differentiation of these five different toxins at the subtype level despite similarities as high as 98% in the case of BoNT/B2 and /B3 [30].

A new method with ultra-high sequence coverage that allowed mass spectrometric differentiation beyond the subtype levels in a complex matrix was recently reported by our laboratory [28]. This work used SDS-PAGE to separate the components of the toxin complex and sequential multiple-enzyme digestion of each gel band. Following LC-MS/MS analysis and database searching, sequence coverage of 90% and above was reported for all seven serotypes of BoNT. Importantly, this technique identified the toxin present in a toxin-contaminated carrot juice sample with sequence coverage of 98.6%, differentiating it from other BoNT/A1 99.9% and 99.6% identical to the toxin variant identified in this sample.

The aforementioned proteomic methods depend upon matching the mass spectral data from a protein of interest to amino acid sequences of known proteins within a database. This could result in mistaken identification if the correct sequence of the protein is not in the database. In 2012, we reported a method to identify novel subtypes or toxin variants of BoNT/B whose amino acid sequences are not in the database [30]. An amino acid substitution database with alteration of each amino acid within the sequence of BoNT/B1 was created and MS/MS data were searched for a possible match. This technique was used to identify five novel amino acid differences in BoNT/B extracted from bacterial culture supernatant and resulted in the identification of a new subtype of BoNT/B, BoNT/B7 [30]. Mass spectrometric results were confirmed with DNA sequencing.

## 3. Qualitative Mass Spectrometric Detection of BoNT Activity

**Figure 4:**
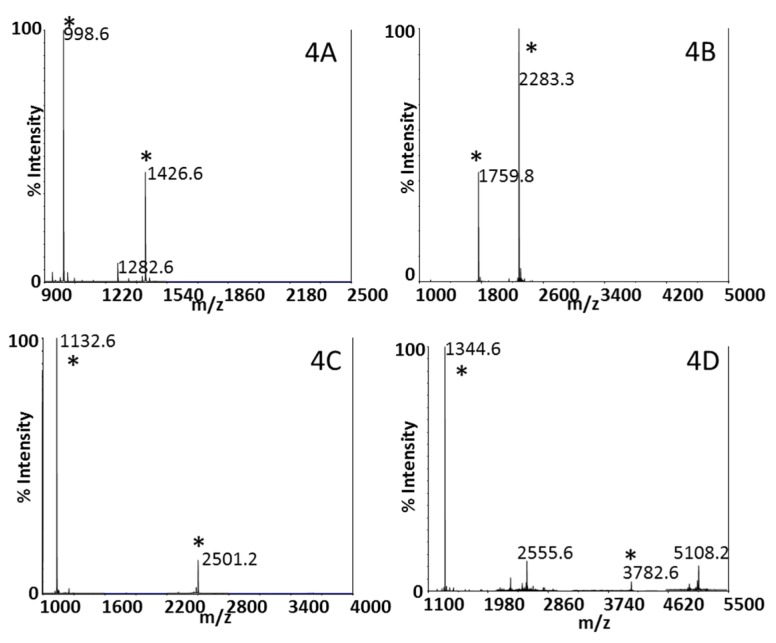
MALDI-TOF mass spectra indicating the presence of (**A**) BoNT/A; (**B**) /B; (**C**) /E; and (**D**) /F. Peptide cleavage products indicating the presence of the neurotoxin are marked with asterisks.

Beyond identification of the presence of BoNT through proteomics methods, mass spectrometry can be used to determine the enzymatic activity of the toxin to offer an accurate measurement of the health threat of BoNT. Our laboratory first reported this method in 2005 with incubation of BoNT with a peptide substrate similar to the toxin’s natural target, SNAP-25 or synaptobrevin-2 [32,33]. Although BoNT/A, /C, and /E cleave SNAP-25 and /B, /D, /F and /G cleave synaptobrevin-2, each serotype of BoNT cleaves in a different location, as seen in Figure 1. Thus, peptide substrates incorporating the different cleavage locations of BoNTs will be cleaved in a serotype-specific fashion. The peptide mixture is analyzed by MALDI-TOF mass spectrometry, which accurately reports the mass of peptides, both cleaved and intact. Detection of peptide cleavage products corresponding to a particular cleavage site indicates the presence of a specific BoNT serotype. Conversely, if the peptide substrate either remains intact or is cleaved in a location other than the toxin-specific site, then that BoNT serotype is absent. The amino acid sequences of the peptide substrates are listed in Table 1, and Figure 4 depicts the cleavage of peptide substrates indicating the presence of BoNT/A, /B, /E, and /F.

**Table 1:** Peptide sequences of the substrates with their observed *m*/*z*. X represents norleucine and hR represents homoarginine.

Peptide	Sequence	*m*/*z* Observed
BoNT/A substrate	Acetyl-RGSNKPKIDAGNQRATRXLGGR-NH_2_	2406
BoNT/B substrate	LSELDDRADALQAGASQFESSAAKLKRKYWWKNLK	4025
BoNT/E substrate	WWWAKLGQEIDTRNRQKDhRIMAKADSNKR-NH_2_	3614
BoNT/F substrate	TSNRRLQQTQAQVDEVVDIMRVNVDKVLERDQKLSELDDRADAL	5111

Additional selectivity ensuring detection and differentiation of BoNT in complex matrices was reported in 2006 where we incorporated an antibody-extraction step preceding the enzymatic reaction [34]. In this work, magnetic beads coated with antibodies to a specific serotype of BoNT were incubated with serum and stool extracts containing BoNT/A, /B, /E, and /F. This technique permitted detection of BoNT/B, /E, and /F in serum at levels below the limit of detection of the mouse bioassay, the traditional method for botulism diagnosis. The limit of detection was somewhat higher in stool extracts due to the protease-rich environment in which proteases endogenous in stool cleaved the peptide substrate intended for BoNT. The use of protease inhibitors improved the limit of detection; however, which was as high as 100 mouse medial lethal dose (mLD_50_) for BoNT/A in stool [34]. A few years later, an additional post-binding bead washing step incorporating 2 M NaCl was reported by our laboratory, allowing for detection of as little 0.5 mLD_50_ of BoNT/A spiked into stool extract [35].

Improvement of the BoNT activity assay included alteration of the amino acid sequence of the peptide substrate used for detection of BoNT/A [36], BoNT/B [37], BoNT/C [38], BoNT/E [39], and BoNT/F [40]. Another improvement included the use of high-affinity, monoclonal antibodies to extract BoNT/A [41] and /B [42] from complex matrices. Use of a high-affinity monoclonal which binds to the heavy chain of the toxin does not inhibit the toxin’s enzymatic activity unlike antibodies which bind to the enzymatic light chain of the toxin. Additionally, the use of high-affinity antibodies allows for more stringent washing of the toxin-bound beads post-extraction as reported previously [36]. Interestingly, attachment of specific monoclonal antibodies was reported to enhance the enzymatic activity of BoNT/B [42]. In combination, these improvements resulted in a limit of detection of 0.5 mLD_50_ of BoNT/A [36] and 0.1 mLD_50_ of BoNT/B [42].

This assay also was reported to detect non-commercially available subtypes of BoNT/A, /B, /E, and /F [43]. Although this activity assay cannot differentiate the subtypes, detection is important as botulism outbreaks can be caused by any subtype of BoNT, many of which are not commercially available yet exist in the environment and in clinical samples. At the time of publication, this method was reported to be able to detect 15 of the 17 known subtypes of BoNT/A, /B, /E, and /F, with BoNT/F7 and /E6 as exceptions. The method has since been reported to detect BoNT/F7 [40] and has also reported the ability to detect two new subtypes: BoNT/B7 [30] and /E9 [44]. All subtypes within a serotype were reported to cleave the peptide substrate in the same location [43] with the exception of BoNT/F5, and this method was also reported for detection of BoNT/F5 [17]. Furthermore, we reported the use of a single, high-affinity monoclonal antibody for extraction of all known BoNT/A, /B, /E, and /F with detection by mass spectrometry, enabling the detection of all BoNT known to cause disease in humans using a single antibody for extraction [31].

## 4. Quantitative Mass Spectrometric Detection of Anthrax Lethal Factor Activity

**Figure 5:**
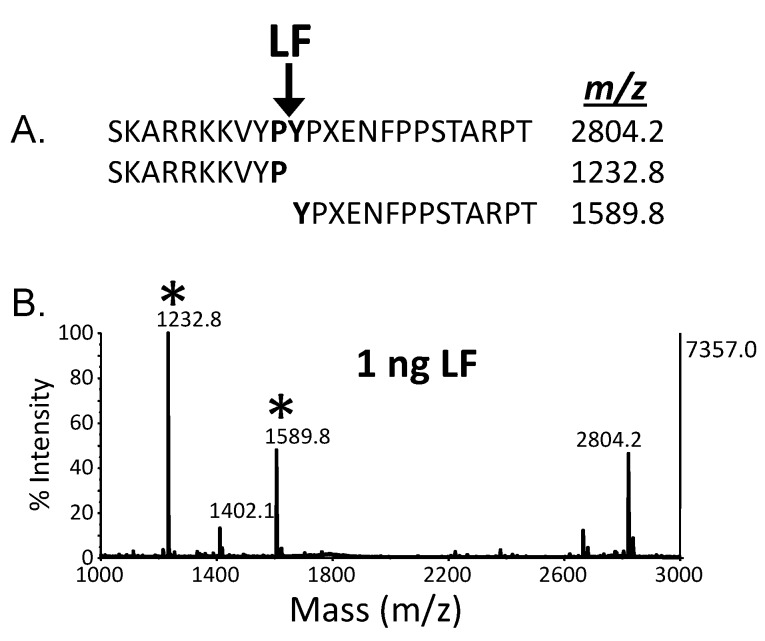
(**B**) MALDI-TOF mass spectrum indicating the presence of anthrax lethal factor; (**A**) Sequence of substrate and cleaved product and expected *m*/*z* of each. Peptide cleavage products indicating the presence of the anthrax lethal factor are marked with asterisks. X = norleucine.

Another example of mass spectrometric detection of enzymatic activity of a protein toxin developed by our laboratory is for detection of anthrax lethal factor [45]. In this work, LF was extracted from serum using murine monoclonal antibodies to the toxin. The captured toxin was then added to a reaction buffer containing an optimized peptide substrate which has a similar amino acid sequence to the portion of MAPKK which is cleaved by anthrax lethal factor. In the presence of the toxin, the peptide substrate was cleaved and similar to the BoNT activity method, the peptide cleavage products were detected by MALDI-TOF mass spectrometry as shown in Figure 5. The intensity of the cleavage products is directly related to the amount of anthrax lethal factor; as the amount of anthrax lethal factor increases, the intensity of the cleavage products also increases in a linear fashion. MALDI-TOF MS quantification was possible through the addition of isotopically-labeled peptide cleavage products which have the same amino acid sequence as the peptide cleavage products yet have a higher mass due to incorporation of isotopically-labeled amino acid residues. These labeled peptide cleavage products were added equally to each sample to serve as an internal standard. Through comparison of the area of the peaks of the native and labeled peptide cleavage products, a determination of the amount of peptide cleavage product produced by LF can be made. Thus, the method for detection of anthrax lethal factor is one which is easily quantifiable, provided that the proper calibrants are used in the analysis.

A limit of detection of 0.05 ng/mL in serum was reported using a 4 h total time for the assay, with detection an order of magnitude lower by extending the assay to a 20 h total time [45]. Quantitative measurements were optimal in the range of 0.05–10 ng/mL using 200 µL of serum. Isotope dilution MALDI-TOF/MS has not traditionally been used for accurate quantification. Therefore, its utility for quantification of lethal factor was verified by comparison to traditional isotope dilution LC-MS/MS quantitative methods [46].

Quantitative measurements of lethal factor are important as this allows a study of toxemia over the time course of infection, yielding a better understanding of anthrax progression. For example, we used this quantitative method to study the kinetics of lethal factor during the course of inhalation anthrax in rhesus macaques [47]. Lethal factor was found to display a triphasic kinetic profile, with low levels at 24 h after anthrax exposure, increasing at 48 h after exposure, declining at 72 h post-exposure, and then increasing again at 96 h post-exposure. Additionally, this method allowed for early diagnosis of inhalation anthrax as it is the only method among four others tested, which reported positive results at the 24 h time point [47].

This method was also used to detect and quantitate lethal factor in serum from suspected naturally-acquired cutaneous anthrax [48]. This report demonstrated the ability of this high sensitivity MS-activity method to detect circulating anthrax toxin secreted from a localized cutaneous lesion. Diagnostic tests focusing on the organism must use tissue or fluid from lesion itself which can be difficult to collect. These tests are also subject to false negatives from antibiotic use. In this study, the organism was detected in lesion samples from only 2 of 9 case-patients (22%) that received antibiotics prior to sample collection whereas lethal factor was detected in the serum of all nine cases (100%). This included two cases that had reported antibiotic use for 7 days. The measurement of lethal factor can also be useful for evaluating the effectiveness of anthrax therapeutics as demonstrated for two inhalation anthrax case-patients [49,50]. During the first days of hospitalization, lethal factor levels declined gradually with antibiotic treatment. When a novel anti-toxin treatment was administered, lethal factor levels declined rapidly, indicating its effectiveness in clearing anthrax toxins in both cases.

In addition to using a MALDI-TOF MS platform for detection of the peptide cleavage products, this method was also developed on the HPLC-ESI-MS/MS for detection of the enzymatic activity of anthrax lethal factor [46]. Many laboratories have triple quadrupole mass spectrometers, so modification of the method to use this different platform enables transfer of this method to a plethora of laboratories. Calibration curves were reported to be linear from 0.05–2.5 ng/mL at the 2 h reaction time, and from 0.005–1 ng/mL at the 18 h reaction time, with a limit of detection of 0.005 ng/mL using a 200 µL sample. 158 serum samples from an animal study were used for comparison of both platforms with matching concentrations of lethal factor obtained, with a standard deviation of ±9%.

## 5. Discussion and Conclusions

Currently, mass spectrometry is poised to become a major tool in the arsenal to correctly diagnose bacterial disease. The FDA has approved two mass spectrometer systems for bacterial identification in human specimens, and other mass spectrometers are expected to follow the same path soon. This technique is expected to revolutionize diagnosis of disease through rapid and precise identification of bacteria causing the disease state, and also has potential to improve treatment and prevention of disease. In select cases, the disease state is caused through exposure not to the bacterium itself, but rather, to a toxin produced by the bacterium. In such cases, it is preferable to identify the presence and function of the toxin itself as the toxin can be present, causing a disease state, even in the absence of bacterial organisms. Two examples of mass spectrometric detection of bacterial protein toxins are presented in this work.

Mass spectrometry can be used to first determine the presence or absence of the toxin. A proteomic study of the toxin in question in combination with database searching has been shown to correctly identify the presence of BoNT even in the midst of complex matrices. This method is able to differentiate the serotype of BoNT and can even further differentiate neurotoxins at the subtype and toxin variant level, allowing for differentiation of proteins which are 99.9% identical in their amino acid sequence. High sequence coverage is critical as some serotypes and subtypes of BoNT have many toxin variants with as little as a single amino acid difference. Such exact differentiation is helpful during epidemiological and forensic investigations which attempt to discover the toxin’s origin and spread during a botulism incident. In this manner, simultaneous botulism outbreaks could be identified as deriving from identical or different sources based upon the subtype or toxin variant present. This differentiation is performed in the absence of the bacterial organism and DNA, and is the only published method which can do so.

However, ascertainment of the presence of BoNT does not necessarily relate to diagnosis of the disease of botulism. Because botulism is caused by intoxication by enzymatically active BoNT, it is critical to determine the enzymatic activity of the BoNT. Such a determination is possible through mass spectrometric measurement of the activity of BoNT upon a peptide substrate which mimics the toxin’s natural target protein. Mass spectrometric detection of a peptide substrate cleaved in a toxin-specific location allows for detection of enzymatically BoNT and differentiation of the serotype. This method has been shown to be more sensitive than the current standard for diagnosis of botulism; the mouse bioassay. The method has been reported to be used in a variety of matrices helpful for diagnosis of botulism such as serum, stool, and bacterial culture supernatants. Furthermore, the method has demonstrated a capacity as a universal detection method for botulism by demonstrating its ability to detect all available subtypes.

A final example of detection of bacterial protein toxin by mass spectrometry is the detection of anthrax lethal factor. This method also relies upon detection of the cleavage of a peptide substrate by mass spectrometry; however, this method utilizes a peptide which mimics the protein target of LF and the method is quantitative. Quantitative measurements allow for a better understanding of the disease state through discovery of the pharmacokinetics of the toxin, and this method was used to discover the triphasic state of lethal factor toxemia. Additionally, quantitative measurements have the potential to impact treatment of anthrax toxemia through understanding the effect of various medical countermeasures.

In conclusion, mass spectrometric measurements of bacterial protein toxins allow for determination of the presence of the toxin and its activity. These measurements can be performed in the presence of complex matrices such as serum, stool, and bacterial culture supernatant. Such measurements allow for proper diagnosis of botulism or anthrax toxemia. Differentiation of the serotype, subtype, and toxin variant of BoNT yields information for epidemiological and forensics investigations whereas quantitation of anthrax lethal factor provides insight into the mechanism of toxemia. These measurements are expected to become more commonplace as mass spectrometers continue to expand into clinical laboratories.
